# Updated guideline for closure of abdominal wall incisions from the European and American Hernia Societies

**DOI:** 10.1093/bjs/znac302

**Published:** 2022-08-26

**Authors:** Eva B Deerenberg, Nadia A Henriksen, George A Antoniou, Stavros A Antoniou, Wichor M Bramer, John P Fischer, Rene H Fortelny, Hakan Gök, Hobart W Harris, William Hope, Charlotte M Horne, Thomas K Jensen, Ferdinand Köckerling, Alexander Kretschmer, Manuel López-Cano, Flavio Malcher, Jenny M Shao, Juliette C Slieker, Gijs H J de Smet, Cesare Stabilini, Jared Torkington, Filip E Muysoms

**Affiliations:** Department of Surgery, Franciscus Gasthuis en Vlietland, Rotterdam, the Netherlands; Department of Hepatic and Digestive diseases, Herlev University Hospital, Copenhagen, Denmark; Department of Vascular Surgery, Manchester University NHS Foundation Trust, Manchester, UK; Mediterranean Hospital of Cyprus, Limassol, Cyprus; Medical School, European University Cyprus, Nicosia, Cyprus; Medical Library, Erasmus MC, Erasmus University Medical Centre, Rotterdam, the Netherlands; Department of Plastic Surgery, University of Pennsylvania Health System, Penn Presbyterian Medical Center, Philadelphia, Pennsylvania, USA; Certified Hernia Center, Wilhelminenspital, Veinna, Austria; Paracelsus Medical, University Salzburg, Salzburg, Austria; Hernia Istanbul®, Hernia Surgery Centre, Istanbul, Turkey; Department of Surgery, University of California San Francisco, San Francisco, California, USA; Department of Surgery, Novant/New Hanover Regional Medical Center, Wilmington, North Carolina, USA; Department of Surgery, Penn State Health Department, Hershey, Pennsylvania, USA; Department of Hepatic and Digestive diseases, Herlev University Hospital, Copenhagen, Denmark; Hernia Center, Vivantes Humboldt-Hospital, Academic Teaching Hospital of Charité University Medicine, Berlin, Germany; Klinikum der Ludwig-Maximillians-Universität München, Munchen, Germany; Janssen Oncology, Los Angeles, CA, USA; Abdominal Wall Surgery Unit, Department of General Surgery, Hospital Universitari Vall d’Hebron, Unviversitat Autònoma de Barcelona, Barcelona, Spain; Department of Surgery, NYU Langone Health/NYU Grossman School of Medicine, New York, New York, USA; Division of Gastrointestinal Surgery, University of Pennsylvania, Philadelphia, Pennsylvania, USA; Department of Surgery, Kantonsspital Baden, Baden, Switzerland; Department of Surgery, Erasmus University Medical Centre, Rotterdam, the Netherlands; Department of Surgery, Policlinico San Martino IRCCS and Department of Surgical Sciences, University of Genoa, Genoa, Italy; Department of Surgery, University Hospital of Wales, Cardiff, UK; Department of Surgery, Maria Middelares Hospital, Ghent, Belgium

## Abstract

**Background:**

Incisional hernia is a frequent complication of abdominal wall incision. Surgical technique is an important risk factor for the development of incisional hernia. The aim of these updated guidelines was to provide recommendations to decrease the incidence of incisional hernia.

**Methods:**

A systematic literature search of MEDLINE, Embase, and Cochrane CENTRAL was performed on 22 January 2022. The Scottish Intercollegiate Guidelines Network instrument was used to evaluate systematic reviews and meta-analyses, RCTs, and cohort studies. The GRADE approach (Grading of Recommendations, Assessment, Development and Evaluation) was used to appraise the certainty of the evidence. The guidelines group consisted of surgical specialists, a biomedical information specialist, certified guideline methodologist, and patient representative.

**Results:**

Thirty-nine papers were included covering seven key questions, and weak recommendations were made for all of these. Laparoscopic surgery and non-midline incisions are suggested to be preferred when safe and feasible. In laparoscopic surgery, suturing the fascial defect of trocar sites of 10 mm and larger is advised, especially after single-incision laparoscopic surgery and at the umbilicus. For closure of an elective midline laparotomy, a continuous small-bites suturing technique with a slowly absorbable suture is suggested. Prophylactic mesh augmentation after elective midline laparotomy can be considered to reduce the risk of incisional hernia; a permanent synthetic mesh in either the onlay or retromuscular position is advised.

**Conclusion:**

These updated guidelines may help surgeons in selecting the optimal approach and location of abdominal wall incisions.

## Teaser

Incisional hernia is a frequent complication of abdominal wall incision and surgical factors contribute to its development. These updated guidelines provide recommendations for surgeons in selecting the approach and location of abdominal wall incisions, and the strategies that might be employed in closing these incisions in adults to minimize the risk of incisional hernia.

## Introduction

Incisional hernias are frequent complications of abdominal wall incisions. A meta-analysis^1^ including over 14 000 patients reported a weighted incidence of 12.8 per cent 2 years after a midline incision, and that one-third of patients with an incisional hernia undergo surgical repair. Recurrence rates after repair of incisional hernia range between 23 and 50 per cent, with increasing rates of complications and re-recurrence after each subsequent failed repair^2^. A reduction in incidence of incisional hernia by 5 per cent was calculated to result in a cost saving of €4 million per year in France^3^. Prevention of incisional hernia is therefore important.

Patient factors contribute to the risk of developing an incisional hernia, including obesity, age, smoking, aneurysmal disease, and wound infections^1,4^. Location of the incision, suture material used, and closure technique are also well known risk factors. In 2015, the European Hernia Society (EHS)^5^ published guidelines on closure of abdominal wall incisions. Since then, several knowledge gaps, as identified by the guidelines committee, have been addressed in systematic reviews and meta-analyses^6–9^. This warranted an update of the EHS guidelines, which was performed in collaboration with the American Hernia Society (AHS).

The objective of this paper was to provide an up-to-date guideline on abdominal wall incisions in adults with the aim of reducing the incidence of incisional hernia. Secondary objectives were to provide recommendations on the prevention of burst abdomen, surgical-site occurrences (SSOs, including wound infection, wound necrosis, wound dehiscence, haematoma, and seroma), and postoperative pain, and improvement in abdominal wall function and cosmesis in patients undergoing abdominal surgery.

## Methods

### Guideline group

The project to update the EHS guidelines was approved by the EHS board and started with a kick-off meeting in December 2020. The AHS joined in August 2021. Between December 2020 and January 2022, four virtual and one in-person meeting were held. A steering group consisting of the first, second, and last author was appointed by the EHS to manage the project. A biomedical information specialist and a certified guideline methodologist were involved in the methodology and search strategy. The guideline group included general surgeons, abdominal wall surgeons, colorectal surgeons, upper gastrointestinal surgeons, a hepatobiliary surgeon, an emergency surgeon, a plastic surgeon, a vascular surgeon, and a urologist. A patient representative was invited to all group meetings, and was involved in prioritizing outcome parameters. A complete list of the members of the group and their responsibilities is available in *Table S1*. Conflicts of interest for each member were recorded transparently at the beginning and completion of the project. The meetings were funded by the EHS. The EHS had no influence on the content of the guidelines. There was no involvement from industry.

### Methodology and literature search

The methodology was same as that used for the 2015 guidelines^5^. The Appraisal of Guidelines for Research and Evaluation (AGREE) instrument was followed^10^. Key questions (KQs) were formulated and translated into PICO (patients—intervention—comparison—outcome) frameworks. KQs were proposed by the coordinators, discussed with the whole group, revised when needed, and approved by the group. For several KQs, separate searches specific to emergency surgery and surgery in obese patients were undertaken. The final list of KQs and PICOs can be found in *Appendix S1*. A biomedical information specialist performed the literature search for all KQs using Medical Subject Headings (MeSH) terms MEDLINE ALL OVID, Emtree terms in Embase.com with terms in the title and/or abstract, and a search in the Cochrane CENTRAL database. This involved a search for systematic reviews and/or meta-analyses on the KQs, and for RCTs published after the search date in the systematic review of a particular question. If a certain KQ could not be answered by up-to-date meta-analyses or systematic reviews of acceptable quality according to the Scottish Intercollegiate Guidelines Network (SIGN) checklists, a second search was performed for all relevant RCTs or observational studies. Case series, case reports, conference abstracts, and expert opinions were excluded. The search for the original guidelines was undertaken on 11 November 2013. For the present update, the literature search started at 2013 for KQs 2–6. As KQ1 was not part of the original guideline, a literature search without time restrictions was undertaken. Papers reporting on any type of abdominal surgery, with any follow-up, and written in English or any language spoken by one of the guideline group members, were included. Exclusion criteria were: articles on patients undergoing hernia repair, operated through incisions not on the ventral abdominal wall (groin or thoracic incisions), natural-orifice surgery or extraction sites, and papers on children or pregnant women. The literature search for this guideline update was last performed on 22 January 2022. The search strategy, including search terms used for KQ1, can be found in *Appendix S2*.

Title and abstract screening of the complete body of evidence from the literature searches was performed by six guideline group members and the evidence summarized for each KQ. Members of the guideline group were divided into six subgroups that evaluated the selected full-text papers on specific KQs. To avoid any conflict of interest, care was taken to ensure that subgroup members did not assess papers that they authored or co-authored. Records were screened by title and abstract by at least two assessors. Full texts were evaluated by at least two assessors independently. Only papers rated as being of acceptable or high quality according to the SIGN checklist^11^ were included, to limit the risk of bias. Any disagreement between assessors was settled by discussion either in the entire group or by a third assessor. References of all papers included in full-text assessment were cross-checked for studies not identified by the literature search. Data from acceptable or high-quality articles were tabulated in summary-of-evidence tables.

The Grading of Recommendations, Assessment, Development and Evaluation (GRADE) approach was used to appraise the papers with respect to the five domains of certainty (risk of bias, imprecision, inconsistency, indirectness, and publication bias) and to generate evidence tables. Evidence for each KQ was then rated according to the GRADE scale, ranging from very low (X000) to high (XXXX)^12^ (*Table S2*). The subgroup proposed the recommendations to the whole guidelines group within an evidence-to-decision framework^12^. Based on the evidence from the literature, clinical experience of the members and patient values, the group reached consensus on recommendations for each KQ when 75 per cent or more of the members agreed. Recommendations were classified as strong or weak in line with GRADE methodology. If there was no evidence for a KQ, or it was of inadequate quality, no recommendation was made. All subgroup members wrote the text of their research question, and these were combined and edited by the first author. Language and style editing was undertaken by a native speaker. The steering group critically revised the manuscript, and the full manuscript was sent to all co-authors for review. Before submission, the manuscript was appraised by five experts, including a methodological expert, and evaluated using the AGREE II instrument^10,13^. The AGREE II instrument assesses the methodological rigour and transparency in which a guideline is developed. The tool is used to calculate a quality score for six domains. The guideline was adjusted according points raised by the AGREE appraisers.

## Results

The PRISMA flow diagram^14^ for the review can be found in *Fig. S1*. A total of 39 papers were included.

### Open and minimally invasive abdominal surgery

#### KQ1a Which approach, minimally invasive or open, should be used in patients undergoing abdominal surgery?

**Table:** 

**KQ1a Which approach, minimally invasive or open, should be used in patients undergoing abdominal surgery?**
**Statement:** There is a decreased risk of both incisional hernia and surgical-site occurrences in patients undergoing laparoscopic operations compared with open operations.
**Recommendation:** Laparoscopic surgery is suggested to be used when safe and feasible to reduce the risk of incisional hernia and surgical-site occurrence.
**Quality of evidence:** XX00 (low)
**Strength of recommendation:** Weak

Abdominal surgery can be performed by a minimally invasive approach, including conventional multiport, robot-assisted or single-incision (SILS) laparoscopic surgery, or by an open approach (laparotomy). One systematic review and meta-analysis^15^, two RCTs^16,17^, and one retrospective review^18^ of an RCT were identified that compared laparoscopic and open surgery for incisional hernia and surgical-site occurrence (SSO) (5549 and 2716 patients respectively from 24 RCTs). There was a significant decrease in incidence of incisional hernia for patients undergoing laparoscopic compared with open surgery (4.3 *versus* 10.1 per cent; *P* < 0.001). In a subgroup of 12 studies comparing total laparoscopic (without extraction sites) *versus* open surgery, the rate of incisional hernia was even lower in the laparoscopic group (0.8 *versus* 16.4 per cent; *P* = 0.001)^15^. The other studies evaluated patients after laparoscopic *versus* open colorectal surgery, and all demonstrated lower rates of incisional hernia in the laparoscopic group^16–18^, but the results were not statistically significant. It was not specified whether hernias occurred at trocar or specimen extraction sites. Wound infection rates were lower for laparoscopic than open surgery (5.0 *versus* 11.4 per cent; *P* < 0.001)^15^.

Overall, there is a paucity of studies that evaluated incisional hernia and SSC rates as the primary outcome. Limitations included the heterogeneity of the studies evaluated, publication bias, imprecision, and indirectness.

#### KQ 1b Which incision should be used in patients undergoing abdominal surgery?

**Table:** 

**KQ1b Which incision should be used in patients undergoing abdominal surgery?** **Statement:** Midline incisions have the highest rate of incisional hernia, including smaller incisions such as specimen extraction sites. There are limited data on surgical-site occurrence, pain, abdominal wall function or cosmesis. **Recommendation:** It is suggested to avoid a midline incision for laparotomies and specimen extraction sites to reduce the risk of incisional hernia. **Quality of evidence:** XX00 (low) **Strength of recommendation:** Weak

In the original guideline^5^, two systematic reviews, including 24 RCTs and over 3700 patients, showed a lower incisional hernia rate after transverse and paramedian laparotomy incisions compared with midline incisions. For this update, no additional studies comparing midline and non-midline laparotomy incisions were found. For specimen extraction sites in laparoscopic surgery, two systematic reviews and meta-analyses, two RCTs, and three cohort studies were found. In an RCT^19^ of midline *versus* transverse incision for specimen extraction after laparoscopic colectomy (165 patients), a transverse incision was associated with a lower incidence of incisional hernia after 30 months of follow up (2 *versus* 15 per cent; *P* = 0.013), but worse cosmesis. Another small RCT of 40 patients was included alongside 16 observational studies in a meta-analysis^20^ of over 6000 patients, and demonstrated an increased risk of incisional hernia at extraction sites for midline compared with non-midline incisions, which included transverse and Pfannenstiel incisions (OR 4.1, 95 per cent c.i. 2.0 to 8.3; *P* < 0.001). Three more recently published cohort studies^21–23^ of over 800 patients looking at extraction sites after colorectal resections confirmed these results, and all reported that midline extraction sites had the highest rate of incisional hernia. A recent systematic review^24^ of nine RCTs, including 1036 patients, compared outcomes of umbilical *versus* epigastric gallbladder extraction during laparoscopic cholecystectomy. No differences in postoperative pain after 24 h or SSO were found. The included RCTs reporting on the incidence of incisional hernias were of insufficient quality to draw meaningful conclusions on this outcome.

### Closure of minimally invasive surgery ports

#### KQ2 Should trocar sites be closed in patients undergoing laparoscopic surgery?

**Table:** 

**KQ2 Should trocar sites be closed in patients undergoing laparoscopic surgery?**
**Statement:** The evidence on trocar-site closure is very limited. The risk of developing a trocar-site hernia is increased for trocar sites of 10 mm or larger, for single-incision laparoscopic surgery (SILS) trocars, and for trocars placed at the umbilical site. There are no robust data supporting fascial closure at the trocar site for prevention of trocar-site hernia, and there are no data on surgical-site complications and pain. There are no data on optimal closure technique or material.
**Recommendation:** It is suggested to suture the fascial defect for trocar sites of 10 mm or larger, especially after SILS and for trocars located at the umbilical site.
**Quality of evidence:** X000 (very low)
**Strength of recommendation:** Weak

The literature search revealed five new publications including a Dutch guideline^25^ on laparoscopy for gynaecologists, a systematic review^26^ on technical risk factors for trocar-site hernias, a systematic review and meta-analysis^27^ of trocar-site closure in bariatric surgery, and two systematic reviews and meta-analyses^28,29^ of SILS. As the Dutch guideline did not include any new studies, it was excluded from this guideline.

Trocar-site hernia appears to be a rare complication of laparoscopic surgery, but the true incidence is probably under-reported. The low-quality systematic review^26^ of technical risk factors for trocar-site hernias confirmed the data used in the original guideline of an overall incidence of trocar-site hernias of 0.1–0.5 per cent, with the highest incidences at the umbilical port site and in 12-mm ports, and the lowest incidence in 5-mm trocars^5,26^. In bariatric surgery, a review^27^ noted an overall trocar-site hernia incidence of 3.2 per cent. This review demonstrated the incidence of trocar-site hernias to be significantly higher in studies that used imaging for their diagnosis than in studies that used clinical examination or no specific follow-up regimen (16.2 *versus* 1.3 per cent respectively). A recent case series^30^ of 79 patients who underwent a laparoscopic gastric sleeve procedure reported a trocar-site hernia rate of 21.5 per cent at the umbilical extraction site, when examined by CT after a mean follow-up of 37 months. This emphasizes the probable underestimation of the true incidence of trocar-site hernias. The review authors attempted to compare the incidence of trocar-site hernias for trocar sites that were closed or not closed after bariatric surgery, and could not detect a difference. However, owing to the heterogeneity of the studies, lack of adequate methodology and duration of follow-up for this outcome, these data were not included in the present guidelines.

Two systematic reviews of SILS focused on cholecystectomies in 2838 patients^29^, and SILS for a variety of surgical procedures in 3340 patients^28^. Both meta-analyses showed an increased risk of incisional hernia after SILS compared with conventional laparoscopy, with an OR of over 2.5. Although the RCTs included in these large meta-analyses were of acceptable methodological quality, the majority of the studies were not sufficiently powered for the outcome incisional hernia. Some studies had insufficient follow-up or a poorly described method of incisional hernia detection. The meta-analysis^29^ of cholecystectomies did not show a difference between SSO or postoperative pain between SILS and conventional laparoscopy. Because SILS requires a larger fascial incision than conventional laparoscopy and carries a higher risk of incisional or trocar-site hernia, these incisions should be closed meticulously, as recommended in the original guidelines^5^.

As no additional evidence regarding trocar size, trocar location or fascial closure was found, the recommendation from the previous guideline on trocar size was not changed.

### Closure of laparotomy incisions

#### KQ3 What is the preferred strategy for closing a laparotomy?

**Table:** 

**KQ3 What is the preferred strategy for closing a laparotomy?**
**Statement:** In the available studies of acceptable quality, no superiority of one specific suture material or continuous *versus* interrupted technique could be shown. The combination of a continuous small-bites suturing technique with a slowly absorbable suture reduces the risk of incisional hernia.
**Recommendation**: A continuous small-bites suturing technique with a slowly absorbable suture is suggested for closure of elective midline incisions.
**Quality of evidence:** XX00 (low)
**Strength of recommendation:** Weak

The literature search identified two large meta-analyses^8,31^ addressing the evidence on suture materials and suture technique for the closure of laparotomies, and two systematic review and meta-analyses^7,32^ of antimicrobial-coated sutures. Additionally, three RCTs of antimicrobial-coated sutures,^33–35^ one^36^ of the small-bites technique, and one^37^ comparing mass with layered closure in transverse incisions published after these systematic reviews and meta-analyses were identified.

In the original guideline^5^, continuous closure of the midline abdominal wall was recommended based on a systematic review that included studies with rapidly absorbable multifilament sutures in an interrupted technique and slowly absorbable or non-absorbable sutures in the continuous group. In the more recent two large meta-analyses^8,31^ of closure techniques, efforts were made to perform an analysis excluding trials in which the comparator arm differed by more than one component. However, many smaller and older studies of moderate to very low quality were included. A Cochrane meta-analysis^31^ included 11 RCTs comparing interrupted and continuous closure techniques with the same suture material: four RCTs including 1195 patients, six RCTs that looked at burst abdomen, and six RCTs on surgical-site infection (SSI) including 4933 patients. There was no difference in the rate of incisional hernia between these techniques (10.9 *versus* 12.7 per cent). No statistically significant difference between the groups was found for burst abdomen and SSI. Postoperative pain was not evaluated. As continuous suturing better distributes tension along the suture line, is faster and leaves less foreign body in the wound, the guidelines group advises the use of continuous over interrupted closure for elective laparotomies.

Three large RCTs^36,38,39^ have investigated the small-bites technique with a total of 1722 patients. The two^38,39^ with 1-year follow-up available, including 1222 patients, showed a reduced incisional hernia rate with the small-bites technique (risk ratio (RR) 0.49, 95 per cent c.i. 0.36 to 0.67). The trials of the small-bites technique are recent and of acceptable quality. However, both RCTs have some methodological shortcomings, one being a single-centre pseudorandomized trial and the other not having radiological imaging for all patients during follow-up. The small-bites technique consists of tissue bites of 5–9 mm from the wound edges to incorporate aponeurosis only, with stitches placed 5 mm apart to ensure adequate distribution of tension, performed in a continuous suturing technique with a slowly absorbable suture material. In all studies, a single-thread suture with a small diameter (USP 2/0) was used on a small needle and aponeurosis was approximated, not pulled together forcefully. Tissue perfusion is highest after low-tension closure, and aponeurotic tissue is less vulnerable to ischaemic damage owing to strangulation from sutures than muscle and fatty tissues^40^. The stitch slackens as soft tissues give way under the suture, allowing the aponeurotic edges to become separated, and consequently an incisional hernia develops. The small-bites technique implies a ratio of suture length to wound length of at least 4 : 1. A suture length to wound length ratio of at least 4 : 1 is achieved by use of an adequate number of stitches, size of stitches, and low tension on the suture, and is associated with a lower incidence of incisional hernia^41,42^. In a combined analysis of the short-term results of the three RCTs^36,38,39^, no difference in the occurrence of burst abdomen or SSC was found. The majority of patients had a BMI below 30 kg/m^2^.

Many RCTs have investigated incisional hernia rate, burst abdomen, and SSI for different suture materials. These relatively small and old studies were combined in a meta-analysis^31^ that took into account only trials comparing different suture materials used with the same continuous or interrupted suturing technique. When the same closure technique was used, this meta-analysis failed to show superiority of one suture material over another for incisional hernia, burst abdomen or SSI.

Antimicrobial-coated sutures were introduced to reduce SSI and are now increasingly being used. No evidence was found for antimicrobial-coated sutures in preventing incisional hernia. Two high-quality systematic reviews and meta-analyses^7,32^ examined the rate of SSI after fascial closure. Both found no difference in SSI with use of antimicrobial-coated slowly absorbable monofilament sutures, but they showed a reduced SSI rate for antimicrobial-coated multifilament fast-absorbing sutures. Since then, three RCTs^33–35^ of antimicrobial-coated suture for fascial closure have been published with contradictory results.

Fascial healing is a complex process that takes over a year to be complete. There is a rapid gain in strength from the eighth postoperative day (beginning of proliferative phase) up to the second month (around the end of collagen synthesis)^43^. After 2 months, the fascia has regained 50 per cent of its original strength^43^. When choosing an absorbable suture for laparotomy closure, the mechanical properties of the suture should be taken into account. Tensile strength decreases over time when absorption takes place. After losing approximately 70–80 per cent of their strength, sutures will not be able to withstand forces on the healing fascia and will break. For multifilament fast-absorbing sutures, only 25 per cent of the original strength remains after 4 weeks, and complete absorption has taken place after 8–10 weeks^44–46^. For monofilament slowly absorbable sutures, more than half of the tensile strength remains after 6 weeks and absorption is completed after 6–8 months^44,47^. Given the reduction in tensile strength of fast-absorbing sutures, these are at high risk of breaking before the fascia has healed well enough. Slowly absorbable and non-absorbable sutures do not differ in terms of the occurrence of incisional hernia and burst abdomen. However, non-absorbable sutures have been linked to prolonged wound pain and suture sinus formation^31^. The guideline group suggests the use of a slowly absorbable suture for closing the midline fascia of the abdominal wall in elective laparotomies.

The data on combined techniques consist mostly of interrupted suturing with a fast-absorbing suture material compared with continuous suturing with a slowly absorbable or non-absorbable suture material. Although systematic reviews reported a reduced rate of incisional hernia with a continuous technique with slowly absorbable or non-absorbable sutures in elective surgery, the analyses were based on studies of limited quality and several studies date from more than 30 years ago^5^. The methodology is poorly described in these trials, with lack of informed consent, risk of selection bias, unclear randomization, insufficient duration and method of follow-up, and missing data on important confounders and secondary outcomes. Imprecision owing to few reported events is another shortcoming. Continuous suturing using the small-bites technique and a slowly absorbable monofilament suture appears to be the best practice based on low-certainty evidence^8,31^. For this reason, the guideline group provided a weak recommendation.

No studies looking specifically at obese patients were found. One RCT comparing continuous and interrupted closure of emergency laparotomies was identified. This trial was assessed as being of unacceptable quality owing to insufficient follow-up (duration and 40 per cent of patients lost to follow-up) and therefore excluded^48^. Two meta-analyses^8,31^ included RCTs with midline and a variety of non-midline laparotomies. Subgroups for closure in non-midline incisions were too small. A recent trial^37^ comparing the early results of mass and layered closure of upper abdominal transverse incisions suggested that layered closure should be preferred. However, it represents the initial results of an ongoing RCT with limited clinical value as yet. In summary, as there are no valid data available for emergency laparotomies, laparotomies in obese patients, or laparotomies performed through a non-midline incision, the principles for elective midline laparotomy closure could be applied.

### Identifying patients with an increased risk of incisional hernia development

#### KQ4 Which patients are at increased risk of incisional hernia development?

Incisional hernia formation results from of a combination of factors, such as patient co-morbidities, genetics, anatomy, health-related behaviours, immunosuppressive medication, surgical technique, soft tissue healing, and SSI. As risk varies significantly across procedures and specialties, there is no universal or standard definition of what constitutes a high-risk patient. Instead, risk relative to a specific procedure and specialty need to be considered.

Incisional hernias are the result of inadequate or impaired early wound healing of the myofascial abdominal wall after surgery. Each of the commonly recognized risk factors for hernia formation inhibits wound healing, including SSI (OR 8.55, 95 per cent c.i. 1.54 to 47.5), diabetes (OR 6.68, 2.02 to 22.0)^49^, smoking (OR 3.93, 1.82 to 8.49)^50^, chronic pulmonary disease (COPD) (HR 2.35, 1.44 to 3.83), obesity (HR 1.74, 1.04 to 2.91)^51^, and immunosuppression (OR 2.5, 1.5 to 4.2)^52^.

Risk stratification tools are available to facilitate identification of patients for preventative strategies such as prophylactic mesh. The HERNIA score was initially evaluated in a prospective study from a single institution^51^ and validated by the same group^53^. The revised HERNIA score, which uses BMI, COPD, incision length, and previous abdominal surgery, may be of use^53^. Another group^54^ evaluated risk factors in over 12 000 patients, and developed and validated a risk model to help predict incisional hernia risk. After more patients had been included, the model was further refined and a risk calculator app developed^55^.

### Prophylactic mesh augmentation

#### KQ5a Is mesh augmentation beneficial for closure of elective laparotomies?

**Table:** 

**KQ5a Is mesh augmentation beneficial for closure of elective laparotomies?**
**Statement:** Mesh augmentation after suture closure of a midline abdominal incision reduces the rate of incisional hernia compared with primary suture closure. Studies do not show an increased risk of surgical-site infection. Data on burst abdomen and postoperative pain are limited. Currently, there are no data on mesh augmentation *versus* primary suture closure of non-midline abdominal incisions.
**Recommendation:** Prophylactic mesh augmentation after elective midline laparotomy can be considered to reduce the risk of incisional hernia.
**Quality of evidence:** XX00 (low)
**Strength of recommendation:** Weak

A systematic review and meta-analysis^9^ and a network meta-analysis^56^ of mesh augmentation of suture closure *versus* primary suture closure alone for midline laparotomies have been published. Another three systematic reviews and meta-analyses^57–59^ were identified, reporting data on incisional hernia and other important outcomes, such as SSI and chronic wound pain. A further meta-analysis^60^ examined prophylactic mesh reinforcement after suture closure *versus* primary suture closure in patients undergoing open abdominal aortic aneurysm (AAA) repair. One more recently published RCT^61^ of mesh augmentation after suture closure *versus* primary suture closure in patients operated for AAA was not included in any meta-analyses.

Twelve RCTs comparing outcomes of mesh augmentation with primary closure in 1815 patients who underwent midline laparotomy were collated in a meta-analysis and trial sequential analysis^9^. Minimum follow-up was 1 year. The risk of incisional hernia was lower in patients who had mesh augmentation closure of a midline laparotomy (RR 0.35, 95 per cent c.i. 0.21 to 0.57)^9^. Similarly, a network meta-analysis^56^ of 17 RCTs with a total of 2763 patients, which compared mesh augmentation in different planes with primary closure of a midline laparotomy, showed a lower risk of incisional hernia in patients with mesh augmentation. Such a benefit of mesh augmentation was consistently reported in three other meta-analyses^57–59^, one^59^ of which included only patients with mesh augmentation in the onlay position. The inclusion criteria in most trials were morbid obesity, AAA or a predefined high-risk score for incisional hernia. Strategies employed to diagnose incisional hernia varied considerably across the studies, from clinical examination alone to cross-sectional imaging. Similarly, there was considerable variation in the indication for laparotomy, type of mesh used, and plane of mesh placement, resulting in statistical between-study heterogeneity. Although some authors explicitly reported a suture to wound length ratio of at least 4 : 1 in the primary closure group, information on type of suture and technique used was limited. Furthermore, the small-bites technique was not used in the RCTs, which may have resulted in overestimation of the treatment effect. Almost half of the RCTs were judged to be at high risk of bias mainly because of selective reporting. Sensitivity analyses excluding studies that were deemed to be at high risk of bias corroborated the results of the primary meta-analysis^9^.

A meta-analysis^9^ of three RCTs (562 patients) comparing onlay mesh augmentation with primary closure of midline laparotomies and four RCTs (560 patients) comparing retromuscular mesh augmentation with primary closure of midline abdominal incisions showed no significant difference in SSI. The network meta-analysis^56^ showed no significant difference in SSI between mesh augmentation and primary suturing.

A meta-analysis^58^ of five RCTs (681 patients) found that the odds of chronic incisional pain was no higher in patients who had mesh augmentation than in those with primary abdominal closure (OR 1.63, 95 per cent c.i. 0.98 to 2.71). This meta-analysis included patients with non-midline laparotomies. Similarly, the network meta-analysis^56^ noted no difference in chronic incisional pain between mesh augmentation and primary closure in a meta-analysis of five RCTs (RR 1.48, 95 per cent c.i. 0.96 to 2.29). Limited data are available on other critical outcomes, such as burst abdomen or mesh infection. Four trials^62–65^ reported the need for partial or complete explantation of 22 of 585 implanted prophylactic meshes owing to infection. Other trials^66–76^ including 786 meshes did not report the need for mesh removal.

Other important aspects of mesh augmentation are the additional costs and operating time. Although the costs of a mesh are added to those of the initial procedure, the costs related to development of an incisional hernia are avoided. An American cost–utility analysis^77^ demonstrated prophylactic mesh augmentation after suture closure of the midline in high-risk patients to be more effective, less costly, and overall more cost-effective than primary suture closure.

#### KQ5b Which type of mesh should be used for prophylactic mesh augmentation?

**Table:** 

**KQ5b Which type of mesh should be used for prophylactic mesh augmentation?**
**Statement:** Prophylactic mesh augmentation with synthetic permanent mesh reduces the incidence of incisional hernia compared with primary closure. Reduction in incisional hernia rate is not proven for absorbable synthetic or biological meshes. There are no studies comparing different types of mesh.
**Recommendation:** It is suggested to use a permanent synthetic mesh for prophylactic mesh augmentation.
**Quality of evidence:** X000 (very low)
**Strength of recommendation:** Weak

There are no studies comparing different types of mesh for prophylactic mesh augmentation. Seventeen RCTs^62–64,66–69,71–76,78–81^ investigated the prophylactic effect of the use of one of the three categories of meshes on incisional hernia development after laparotomy: absorbable synthetic mesh, non-absorbable synthetic mesh, and biological mesh. Twelve trials^62–64,67,69,73–76,78–80^ used non-absorbable synthetic mesh, whereas two^72,81^ used fast-absorbing synthetic mesh and three^66,68,71^ used a biological mesh. All trials^62–64,67,69,73–76,78^ comparing non-absorbable synthetic mesh augmentation with a suture-only technique reported a significant reduction in incisional hernia. No benefit of fast-absorbing synthetic mesh augmentation was shown in terms of burst abdomen or incisional hernia rates^72,81^. Three studies^66,68,71^ of the prophylactic effect of a biological mesh reported different results. One study^68^ found no difference in the rate of incisional hernia at 2 years’ follow-up in 380 patients, whereas the other two trials^66,71^, including 132 patients, noted lower rates of incisional hernia in patients treated with biological mesh augmentation. Data on adverse effects of mesh augmentation are also divergent. Four trials^62–65^ reported the need for partial or complete explantation of non-absorbable synthetic meshes. No mesh explantation was reported in trials of absorbable or biological meshes.

#### KQ5c Which abdominal plane should be used for prophylactic mesh augmentation?

**Table:** 

**KQ5c Which abdominal plane should be used for prophylactic mesh augmentation?**
**Statement:** Data concerning prophylactic mesh implantation in different anatomical planes are limited. Onlay and retromuscular mesh implantation seem safe and effective in incisional hernia prevention.
**Recommendation:** When using a prophylactic mesh, onlay or retromuscular implantation is suggested.
**Quality of evidence:** X000 (very low)
**Strength of recommendation:** Weak

Only one RCT^65,78^ has compared onlay *versus* retromuscular prophylactic mesh placement. The 2-year incidence of incisional hernia was comparable after onlay and retromuscular mesh reinforcement (13 *versus* 18 per cent). The risk of seroma formation was significantly increased after onlay mesh placement^65^. However, this did not translate into more interventions or readmissions. The association between prophylactic mesh plane and infectious complications was assessed in a more recently published update of this RCT^82^. Such complications occurred in 17.6 per cent in the onlay group and 10.3 per cent in the retromuscular group (*P* = 0.042). The mesh could remain *in situ* in 77 per cent of patients with an infectious complication,

In a network meta-analysis^56^, prophylactic onlay (RR 0.24, 95 per cent c.i. 0.12 to 0.46) and retromuscular mesh implantation (RR 0.32, 0.16 to 0.66) were associated with a significantly lower risk of incisional hernia than primary suture closure. The number needed to treat was 4 and 5 respectively. Onlay mesh placement was associated with a significantly higher risk of seroma than primary suture closure (RR 2.21, 1.44 to 3.39). Comparing different mesh implantation planes, all except the retromuscular location had a higher risk of wound infection than primary closure, but the finding was not significantly different^56^. Subgroup analysis in another meta-analysis^9^ showed a reduced risk of incisional hernia with both the onlay and retromuscular mesh augmentation techniques compared with primary abdominal wall closure alone. Two RCTs^63,79^ compared intraperitoneal synthetic mesh placement *versus* primary closure alone. Although a reduction in the incidence of incisional hernia was noted, the guideline group does not advise implanting a synthetic mesh prophylactically in the intraperitoneal space, given the increased risk of adhesive complications. Retromuscular mesh implantation is a technique that not all surgical specialists master. Although onlay implantation is associated with an increased risk of SSO, it is easy to perform and, where future incisional hernia repair is required, the retromuscular plane remains intact.

#### KQ5d Is mesh augmentation beneficial during closure of emergency laparotomies?

**Table:** 

**KQ5d Is mesh augmentation beneficial during closure of emergency laparotomies?**
**Statement:** Data on mesh augmentation in emergency midline laparotomy are heterogeneous and limited.
**Recommendation:** No recommendation can be made regarding prophylactic mesh augmentation in emergency laparotomy.
**Quality of evidence:** X000 (very low)
**Strength of recommendation:** –

Mesh augmentation in the context of emergency surgery is important as these patients often have impaired physiology and risk factors for fascial dehiscence and incisional hernia development^48^. There are concerns about mesh infection, particularly in a contaminated field.

Four RCTs^71,80,83–85^ have compared mesh augmentation with primary closure in emergency laparotomies. One trial^83^ of intraperitoneal implantation of a biological mesh in 48 patients was closed prematurely owing to a higher incidence of mesh-related reintervention in the prophylactic mesh group. Mesh-related abdominal wall complications included non-integration of mesh into the abdominal wall and mesh infection.

In two other RCTs^71,84^, including 100 and 200 patients, abdominal wall closure was done with a 3-cm wide strip of biosynthetic mesh implanted retromuscularly^71^ or a 4-cm wide strip of synthetic mesh implanted in the retromuscular position^84^. The incidence of incisional hernia on imaging was higher in the suture group compared with the mesh group (22 *versus* 6 per cent, and 21 *versus* 6 per cent respectively) at 2 years’ follow-up. No difference was found in 30-day morbidity between mesh augmentation and suture closure alone^71,84^. Another RCT^80^ compared the small-bites technique with or without onlay synthetic mesh augmentation. However, only 30-day results were available, which showed a decreased fascial dehiscence rate in the mesh group (0 *versus* 13.5 per cent). SSOs were similar in both groups, but two patients needed partial mesh explantation. No data from this trial are available on long-term incisional hernia rate.

Overall, the quality of the evidence investigating potential benefits of mesh augmentation in an emergency setting is considered very low mainly owing to insufficient small sample sizes and inconsistency because of different indications for laparotomy, different types of implanted mesh, different planes of implantation, and different methods for diagnosis of incisional hernia. More research is needed to draw definitive conclusions on potential benefits of mesh augmentation in patients undergoing emergency midline laparotomy and to identify subgroups of patients who might benefit from prophylactic mesh placement.

### Postoperative care

#### KQ6 Are postoperative abdominal binders advantageous after open abdominal surgery?

**Table:** 

**KQ6 Are postoperative abdominal binders advantageous after open abdominal surgery?**
**Statement:** There are no data on burst abdomen, incisional hernia or surgical-site occurrences related to the use of postoperative binders after open abdominal surgery. There are limited data to suggest that abdominal binders reduce postoperative pain, without compromising pulmonary function.
**Recommendation:** No recommendation can be made for or against the use of postoperative binders owing to the lack of data on their effect on incisional hernia or burst abdomen.
**Quality of evidence:** X000 (very low)
**Strength of recommendation:** –

Since the previous guideline, three systematic reviews and meta-analyses^86–88^ of the use of abdominal binders have been published, all including a different set of RCTs and observational studies. One systematic review^86^ included eight studies, of which were four RCTs, comprising a total of 578 patients. No effect on postoperative pain was found. Use of abdominal binders did not compromise pulmonary function. The other two meta-analyses, including 10 (968 patients)^87^ and five (281 patients)^88^ RCTs reported a reduction in postoperative pain with abdominal binders and no negative effect on pulmonary function. Abdominal binders can be used after open abdominal surgery, but their costs, acceptance by patients, and potential risks should be taken into account. No recommendations can be given in terms of SSO, burst abdomen, and incisional hernia formation as there are a lack of data on these outcomes.

#### KQ7 Is restriction of activity advantageous after open abdominal surgery?

**Table:** 

**KQ7 Is restriction of activity advantageous after open abdominal surgery?**
**Statement:** There are very limited data on restriction of activity after open abdominal surgery.
**Recommendation:** No recommendation on restriction of activity after open abdominal surgery can be made owing to lack of evidence.
**Quality of evidence:** –
**Strength of recommendation:** –

Only one systematic review^89^ on the restriction of activity after abdominal surgery has been published. This included 22 studies, incorporating three RCTs and several opinion articles. No comparative prospective trials on pulmonary function, SSO, burst abdomen, and incisional hernia formation were included. Therefore, no recommendations can be given on the restriction of postoperative physical activities owing to lack of studies. An expert survey^90^ of recommendations regarding postoperative strain and physical labour after abdominal surgery was undertaken during the 41st Annual International Congress of the EHS. Four weeks of no physical strain after laparotomy was considered appropriate by a majority of the experts.

### External appraisal

The guideline was appraised externally by four surgeons (reviewers 1–4) and a member of the AGREE Collaboration (reviewer 5) not involved in guideline development with the AGREE II instrument^10,13^. The assessment of the guideline per domain by the reviewers can be found in *Fig. S2*. The overall rating of the quality of the guideline was good (87 per cent) and all would recommend this guideline for use. AGREE II considers a guideline high quality if it has a score over 70 per cent. Of these six domains, five scored over 70 per cent (72–89 per cent); the domain of applicability scored below this threshold (43 per cent). The reviewers were missing practical advice to help surgeons implement these guidelines into their practice, possible barriers and the costs of adding time to a procedure when implementing recommendations, and the costs and availability of prophylactic mesh augmentation of the abdominal wall. These considerations were used to add a paragraph on implementation to the discussion section. Furthermore, after the AGREE II appraisal, the methodology section was improved by explaining in more detail how the GRADE methodology was used during the guideline process and recommendations were formulated.

## Discussion

### Limitations

These updated guidelines summarize the studies of abdominal wall closure in adults undergoing abdominal surgery. The major limitation of this guideline reflects the limited quantity and/or quality of the studies available to answer the KQs. This makes it impossible to formulate strong recommendations for any of the KQs according to the GRADE methodology^12^. The majority of studies reported on the incidence of burst abdomen, SSI, and incisional hernia. Patient-reported outcomes, such as pain, abdominal wall function and cosmesis, were reported rarely. Data were limited or lacking for emergency surgery and surgery in obese patients, and recommendations for these patients could not be made. Although the guidelines group aimed to represent all stakeholders and surgical specialties, it would have benefited from participation of a gynaecological surgeon and physiotherapist. Care was taken to create subgroups without group members who authored a paper relevant to the KQ or with other conflicts of interest. However, all group members use meshes and sutures, and are involved in hernia prevention, which might have influenced appraisal of the evidence and formulation of recommendations. Efforts were made to have active patient participation, but unfortunately not all group meetings had patient representation. A patient representative critically reviewed the guidelines and their valuable comments were included.

### Implementation

To aid dissemination and implementation, the guideline will be presented at international and national conferences, and summaries will be written in different languages for national journals. Implementation of standard closure techniques in elective and emergency laparotomies has been described in detail, and shown to reduce the incidence of incisional hernia and burst abdomen^91–94^. Before implementation of the recommendations, surgeons may need some extra education and/or training in the techniques. Monitoring of this process, complications, and feedback are important. Surgical procedures can be performed in different positions (such as lateral) and on patients in special conditions (for example, pregnancy), which require special considerations when choosing location of incision, approach, and closure techniques. For these reasons, separate papers on the applicability of the guidelines specifically for urological, gynaecological and vascular procedures and patients will be written. Another barrier to implementation of these guidelines may be the added costs of, and time taken, for a surgical procedure, especially in low- and middle-income countries. However, reduction in the incidence of incisional hernia has been proven to reduce healthcare costs^3^, and even the use of prophylactic mesh has been calculated to be more cost-effective than suture closure alone^77^. Given the additional studies identified for this update, it is planned to update these guidelines 5 years after publication using the GRADE methodology.

### Knowledge gaps

The guideline group discussed the knowledge gaps and proposed some research areas that could improve the evidence for several KQs. The lack of patient-reported outcomes, such as incision pain, abdominal wall function and cosmesis, was acknowledged and these should be included in future studies. Of particular interest will be investigating the effect of different size and location of abdominal wall incisions on cosmesis and abdominal wall function. For this guideline, evidence was also searched for patients with an increased risk of complications, such as those undergoing emergency surgery, obese patients, and immunocompromised individuals. Unfortunately, studies in these groups were lacking. Furthermore, most studies comparing minimally invasive and open approaches did not have sufficient power, or adequate method or duration of follow-up, to evaluate abdominal wall complications such as incisional hernia. With the rise in robotically assisted minimally invasive procedures, studies comparing these procedures with conventional laparoscopy will be of interest. The force on the abdominal wall from the robotic system might influence postoperative outcomes such as pain and trocar-site hernia formation. Disappointingly, most trials have provided insufficient information on suturing technique or the suture length to wound length ratio. Although several high-quality RCTs of prophylactic mesh augmentation have been conducted, none of these compared prophylactic mesh augmentation with primary closure using the small-bites technique. Data on slowly absorbable meshes for prophylactic mesh augmentation were not lacking.

## Supplementary Material

znac302_Supplementary_Data
