# Erratum to: Updated guideline for closure of abdominal wall incisions from the European and American Hernia Societies

**DOI:** 10.1093/bjs/znac412

**Published:** 2022-11-10

This is an erratum to: Eva B Deerenberg, Nadia A Henriksen, George A Antoniou, Stavros A Antoniou, Wichor M Bramer, John P Fischer, Rene H Fortelny, Hakan Gök, Hobart W Harris, William Hope, Charlotte M Horne, Thomas K Jensen, Ferdinand Köckerling, Alexander Kretschmer, Manuel López-Cano, Flavio Malcher, Jenny M Shao, Juliette C Slieker, Gijs H J de Smet, Cesare Stabilini, Jared Torkington, Filip E Muysoms, Updated guideline for closure of abdominal wall incisions from the European and American Hernia Societies, *British Journal of Surgery*, 2022;, znac302, https://doi.org/10.1093/bjs/znac302

In the originally published version, affiliations of some of the authors were incorrect in the HTML version. All affiliations in the PDF version were correct.

These errors have now been corrected.

