# Supplementary material for: Updated guideline for closure of abdominal wall incisions from the European and American Hernia Societies
Source: Br J Surg. 2022 Aug 26;109(12):1239–50. doi: 10.1093/bjs/znac302 (PMC10364727; doi:10.1093/bjs/znac302)
Supplement: znac302_Supplementary_Data [file znac302_supplementary_data.docx]

**Table S1. List of guideline group members and responsibilities**

| Name | Discipline | Institution | Location | Role |
| --- | --- | --- | --- | --- |
| E.B. (Eva) Deerenberg | Colorectal and abdominal wall surgeon | Franciscus Gasthuis en Vlietland | Rotterdam, The Netherlands | - Steering group - Design of literature searches - Screening of titles and abstracts - Literature quality assessment and data interpretation for paragraph on approaches and incisions - Drafting manuscript |
| N.A. (Nadia) Henriksen | Upper GI and abdominal wall surgeon | Herlev University Hospital | Copenhagen, Denmark | - Steering group - Screening of titles and abstracts - Literature quality assessment and data interpretation for paragraph on laparoscopic port closure - Drafting manuscript |
| F.E. (Filip) Muysoms | General and abdominal wall surgeon | AZ Maria Middelares | Ghent, Belgium | - Steering group - Screening of titles and abstracts - Literature quality assessment and data interpretation for paragraph on laparoscopic port closure - Drafting manuscript |
| W.M. (Wichor) Bramer | Biomedical information specialist/ methodologist | Medical Library Erasmus MC | Rotterdam, The Netherlands | - Performed literature searches - Critical revision of manuscript |
| S.A. (Stavros) Antoniou | General surgeon | Mediterranean Hospital of Cyprus  Medical School, European University Cyprus | Limassol, Cyprus  Nicosia, Cyprus | - Certified guideline methodologist - Literature quality assessment and data interpretation for paragraph on laparotomy closure - Critical revision of manuscript |
| G.H.J. (Gijs) de Smet | Medical doctor | Erasmus MC University Medical Center | Rotterdam, The Netherlands | - Design of literature searches - Screening of titles and abstracts - Literature quality assessment and data interpretation for paragraph on postoperative care - Critical revision of manuscript |
| G.A. (George) Antoniou | Vascular surgeon | Manchester University NHS Foundation Trust | Manchester, UK | - Literature quality assessment and data interpretation for paragraph on prophylactic mesh augmentation - Critical revision of manuscript |
| N. (Nicola) Dames | Patient representative | - | Glasgow, Scotland | - Adviser on importance of outcome parameters - Critical revision of manuscript |
| J. (John) Fischer | Plastic surgeon | University of Pennsylvania Health System, Penn Presbyterian Medical Center | Philadelphia, PA, USA | - Literature quality assessment and data interpretation for paragraph on risk factors for incisional hernia development - Critical revision of manuscript |
| R.H. (Rene) Fortelny | Abdominal wall surgeon | Certified Hernia Center, Wilhelminenspital  Paracelsus Medical University Salzburg | Vienna, Austria  Salzburg, Austria | - Literature quality assessment and data interpretation for paragraph on prophylactic mesh augmentation - Critical revision of manuscript |
| H. (Hakan) Gök | Abdominal wall surgeon | Hernia Istanbul®, Hernia Surgery Center | Istanbul, Turkey | - Literature quality assessment and data interpretation for paragraph on postoperative care - Critical revision of manuscript |
| H.W. (Hobart) Harris | Hepato-billiary and abdominal wall surgeon | University of California San Francisco | San Francisco, CA, USA | - Literature quality assessment and data interpretation for paragraph on risk factors for incisional hernia development - Critical revision of manuscript |
| W. (William) Hope | Gastrointestinal and abdominal wall surgeon | Novant/New Hanover Regional Medical Center | Wilmington, NC, USA | - Literature quality assessment and data interpretation for paragraph on risk factors for incisional hernia development - Critical revision of manuscript |
| C.M. (Charlotte) Horne | Mininimally invasive, bariatric and abdominal wall surgeon | Penn State Health Department | Hershey, PA, USA | - Literature quality assessment and data interpretation for paragraph on approaches and incisions - Critical revision of manuscript |
| T.K. (Thomas Korgaard) Jensen | Emergency general surgeon | Herlev University Hospital | Copenhagen, Denmark | - Screening of titles and abstracts - Literature quality assessment and data interpretation for paragraph on prophylactic mesh augmentation - Critical revision of manuscript |
| F. (Ferdinand) Köckerling | General and abdominal wall surgeon | Hernia Center and  Vivantes Humboldt-Hospital | Berlin, Germany | - Literature quality assessment and data interpretation for paragraph on laparoscopic port closure - Critical revision of manuscript |
| A. (Alexander) Kretschmer | Urologist | Klinikum der Ludwig-Maximillians-Universität München  Janssen Oncology | München, Germany  Los Angeles, CA, USS | - Literature quality assessment and data interpretation for paragraph on approaches and incisions - Critical revision of manuscript |
| M. (Manuel) López-Cano | General and abdominal wall surgeon | Hospital Universitari Vall d’Hebron, Unviversitat Autònoma de Barcelona | Barcelona, Spain | - Literature quality assessment and data interpretation for paragraph on laparotomy closure - Critical revision of manuscript |
| F. (Flavio) Malcher | General and abdominal wall surgeon | NYU Langone Health/NYU Grossman School of Medicine | New York, NY, USA | - Literature quality assessment and data interpretation for approaches and incisions - Critical revision of manuscript |
| J.M. (Jenny) Shao | Mininimally invasive, foregut and abdominal wall surgeon | University of Pennsylvania | Philadelphia, PA, USA | - Literature quality assessment and data interpretation for paragraph on approaches and incisions - Critical revision of manuscript |
| J.C. (Juliette) Slieker | General surgeon | Kantonsspital Baden | Baden, Switzerland | - Screening of titles and abstracts - Literature quality assessment and data interpretation for paragraph on laparotomy closure - Critical revision of manuscript |
| C. (Cesare) Stabilini | General surgeon | Policlinico San Martino IRCCS, University of Genoa | Genoa, Italy | - Literature quality assessment and data interpretation for paragraph on prophylactic mesh augmentation - Critical revision of manuscript |
| J. (Jared) Torkington | Colorectal Surgeon | University Hospital of Wales | Cardiff, UK | - Literature quality assessment and data interpretation for paragraph on approaches and incisions - English language editing - Critical revision of manuscript |

**Table S2.** Quality of the body of evidence according to GRADE([12](#_ENREF_12)).

| Quality of evidence according to GRADE | High | XXXX | We are very confident that the estimate of effect lies close to the true effect for this outcome. |
| --- | --- | --- | --- |
|  | Moderate | XXX0 | We are moderately confident that the estimate of effect lies close to the true effect for this outcome. |
|  | Low | XX00 | We have limited confidence that the estimate of effect lies close to the true effect for this outcome. |
|  | Very low | X000 | We have no evidence, we are unable to estimate an effect, or we have no confidence in the estimated of the effect for this outcome. |

**Figure S1.** PRISMA flow diagram([14](#_ENREF_14))

Records removed *before screening*:

- Duplicate records removed (n = 1591)

Records identified from databases (n = 4665)

- Pubmed (n = 2234)
- Embase (n = 1944)
- Cochrane (n = 487)

**Identification**

Records screened by title and abstract

(n = 3074)

Records excluded

(n = 2963)

**Screening**

Reports sought for retrieval

(n = 111 )

Reports not retrieved

(n = 2)

Full-text reports assessed for eligibility

(n = 109)

Reports excluded

(n = 70)

Papers included (n = 39)

- Key question 1 (n = 11)
- Key question 2 (n = 4)
- Key question 3 (n = 9)
- Key question 5 (n = 11)
- Key question 6 (n = 3)
- Key question 7 (n = 1)

**Included**

**Figure S2.** External appraisal of guideline by five reviewers using the AGREE II instrument.

The sum of the items in the domain are presented as percentage of maximum score.

**Appendix S1. Final list of key questions and PICO’s.**

KQ1 Which approach to entering the abdominal cavity and location of incisions should be used in patients undergoing abdominal surgery?

PICO 1.1 Open and laparoscopic surgery

| **P**atient | Adults that underwent abdominal surgery |
| --- | --- |
| **I**ntervention | Laparoscopic approach |
| **C**omparison | Open approach |
| **O**utcome | Burst abdomen, incisional hernia, SSO, cosmetic results and pain |

PICO 1.2 Single-incision/single-port and multi-port laparoscopic surgery

| **P**atient | Adults that underwent laparoscopic abdominal surgery |
| --- | --- |
| **I**ntervention | Single-incision/single-port laparoscopic surgery (SILS) |
| **C**omparison | Multi-port laparoscopic surgery |
| **O**utcome | Burst abdomen, incisional hernia, SSO, cosmetic results and pain |

PICO 1.3 Laparotomy incisions

| **P**atient | Adults that underwent surgery through an abdominal wall incision |  |
| --- | --- | --- |
| **I**ntervention | Non-midline incision |  |
| **C**omparison | Midline incision |  |
| **O**utcome | Burst abdomen, incisional hernia, SSO, cosmetic results and pain |  |

PICO 1.4 Specimen extraction sites

| **P**atient | Adults that underwent laparoscopic surgery and needed an abdominal wall incision for specimen extraction |  |
| --- | --- | --- |
| **I**ntervention | Non-midline incision |  |
| **C**omparison | Midline incision |  |
| **O**utcome | Burst abdomen, incisional hernia, SSO, cosmetic results and pain |  |

PICO 1.5 Trocar placement

| **P**atient | Adults that underwent laparoscopic surgery |  |
| --- | --- | --- |
| **I**ntervention | Non-midline incision |  |
| **C**omparison | Midline incision |  |
| **O**utcome | Burst abdomen, incisional hernia, SSO, cosmetic results and pain |  |

PICO 1.6 Blunt versus bladed trocars

| **P**atient | Adults that underwent laparoscopic surgery |
| --- | --- |
| **I**ntervention | Bladed trocars |
| **C**omparison | Blunt trocars |
| **O**utcome | Incisional hernia |

KQ2 Should trocar sites be closed in patients undergoing laparoscopic surgery?

PICO 2.1 Small trocars (≤5mm)

| **P**atient | Adults that underwent laparoscopic surgery with a trocar ≤5mm |
| --- | --- |
| **I**ntervention | Closure of fascia at trocar site |
| **C**omparison | Not closing fascia at trocar site |
| **O**utcome | Incisional hernia, SSO, pain |

PICO 2.2 Medium trocars (6-9 mm)

| **P**atient | Adults that underwent laparoscopic surgery with a trocar 6-9mm |
| --- | --- |
| **I**ntervention | Closure of fascia at trocar site |
| **C**omparison | Not closing fascia at trocar site |
| **O**utcome | Incisional hernia, SSO, pain |

PICO 2.3 Large trocars (≥10mm)

| **P**atient | Adults that underwent laparoscopic surgery with a trocar ≥10mm |
| --- | --- |
| **I**ntervention | Closure of fascia at trocar site |
| **C**omparison | Not closing fascia at trocar site |
| **O**utcome | Incisional hernia, SSO, pain |

PICO 2.4 Small trocars (≤5mm), suture material

| **P**atient | Adults that underwent laparoscopic surgery with a trocar ≤5mm |
| --- | --- |
| **I**ntervention | Closure of fascia with slow-absorbable suture (polydioxanone) |
| **C**omparison | Closure of fascia with fast-absorbable suture (polyglactine) |
| **O**utcome | Incisional hernia, SSO, pain |

PICO 2.5 Medium trocars (6-9 mm), suture material

| **P**atient | Adults that underwent laparoscopic surgery with a trocar 6-9mm |
| --- | --- |
| **I**ntervention | Closure of fascia with slow-absorbable suture (polydioxanone) |
| **C**omparison | Closure of fascia with fast-absorbable suture (polyglactine) |
| **O**utcome | Incisional hernia, SSO, pain |

PICO 2.6 Large trocars (≥10mm), suture material

| **P**atient | Adults that underwent laparoscopic surgery with a trocar ≥10mm |
| --- | --- |
| **I**ntervention | Closure of fascia with slow-absorbable suture (polydioxanone) |
| **C**omparison | Closure of fascia with fast-absorbable suture (polyglactine) |
| **O**utcome | Incisional hernia, SSO, pain |

KQ2 – obesity - How should trocar sites be closed in **obese** patients undergoing laparoscopic surgery?

PICO 2.8 Small trocars (≤5mm)

| **P**atient | Obese adults that underwent laparoscopic surgery with a trocar ≤5mm |
| --- | --- |
| **I**ntervention | Closure of fascia at trocar site |
| **C**omparison | Not closing fascia at trocar site |
| **O**utcome | Incisional hernia, SSO, pain |

PICO 2.9 Medium trocars (6-9 mm)

| **P**atient | Obese adults that underwent laparoscopic surgery with a trocar 6-9mm |
| --- | --- |
| **I**ntervention | Closure of fascia at trocar site |
| **C**omparison | Not closing fascia at trocar site |
| **O**utcome | Incisional hernia, SSO, pain |

PICO 2.10 Large trocars (≥10mm)

| **P**atient | Obese adults that underwent laparoscopic surgery with a trocar ≥10mm |
| --- | --- |
| **I**ntervention | Closure of fascia at trocar site |
| **C**omparison | Not closing fascia at trocar site |
| **O**utcome | Incisional hernia, SSO, pain |

PICO 2.11 Small trocars (≤5mm), suture material

| **P**atient | Obese adults that underwent laparoscopic surgery with a trocar ≤5mm |
| --- | --- |
| **I**ntervention | Closure of fascia with slow-absorbable suture (polydioxanone) |
| **C**omparison | Closure of fascia with fast-absorbable suture (polyglactine) |
| **O**utcome | Incisional hernia, SSO, pain |

PICO 2.12 Medium trocars (6-9 mm), suture material

| **P**atient | Obese adults that underwent laparoscopic surgery with a trocar 6-9mm |
| --- | --- |
| **I**ntervention | Closure of fascia with slow-absorbable suture (polydioxanone) |
| **C**omparison | Closure of fascia with fast-absorbable suture (polyglactine) |
| **O**utcome | Incisional hernia, SSO, pain |

PICO 2.13 Large trocars (≥10mm), suture material

| **P**atient | Obese adults that underwent laparoscopic surgery with a trocar ≥10mm |
| --- | --- |
| **I**ntervention | Closure of fascia with slow-absorbable suture (polydioxanone) |
| **C**omparison | Closure of fascia with fast-absorbable suture (polyglactine) |
| **O**utcome | Incisional hernia, SSO, pain |

KQ3A What is the preferred strategy to close an **elective** midline laparotomy?

PICO 3.1 Closure technique in midline incision, interrupted vs continuous

| **P**atient | Adults that underwent surgery through a midline incision |
| --- | --- |
| **I**ntervention | Interrupted sutures technique |
| **C**omparison | Continuous closure technique |
| **O**utcome | Burst abdomen, incisional hernia, SSO and pain |

PICO 3.2 Closure technique in midline incision, small bites vs large bites technique

| **P**atient | Adults that underwent surgery through a midline incision |
| --- | --- |
| **I**ntervention | Small bites |
| **C**omparison | Large bites |
| **O**utcome | Burst abdomen, incisional hernia, SSO and pain |

PICO 3.3 Suture material in midline incision

| **P**atient | Adults that underwent surgery through a midline incision |
| --- | --- |
| **I**ntervention | Fast-absorbable suture material |
| **C**omparison | Slowly-absorbable suture material |
| **O**utcome | Burst abdomen, incisional hernia, SSO and pain |

PICO 3.4 Suture material in midline incision

| **P**atient | Adults that underwent surgery through a midline incision |
| --- | --- |
| **I**ntervention | Non-absorbable suture material |
| **C**omparison | Slowly-absorbable suture material |
| **O**utcome | Burst abdomen, incisional hernia, SSO and pain |

PICO 3.5 Suture material in midline incision

| **P**atient | Adults that underwent surgery through a midline incision |
| --- | --- |
| **I**ntervention | Antimicrobial impregnated sutures |
| **C**omparison | Not impregnated sutures |
| **O**utcome | Burst abdomen, incisional hernia, SSO |

PICO 3.6 Suture material in midline incision

| **P**atient | Adults that underwent surgery through a midline incision |
| --- | --- |
| **I**ntervention | Monofilament sutures |
| **C**omparison | Multifilament sutures |
| **O**utcome | Burst abdomen, incisional hernia, SSO |

PICO 3.7 Interrupted absorbable vs continuous non/slowly-absorbable

| **P**atient | Adults that underwent surgery through a midline incision |
| --- | --- |
| **I**ntervention | Interrupted stitches with fast-absorbable sutures |
| **C**omparison | Continuous stitches with slowly/non-absorbable sutures |
| **O**utcome | Burst abdomen, incisional hernia, SSO |

KQ3 – emergency - What is the preferred suture type and closure technique for an **emergency** midline laparotomy?

PICO 3.8 Closure technique in midline incision, interrupted vs continuous

| **P**atient | Adults that underwent emergency surgery through a midline incision |
| --- | --- |
| **I**ntervention | Interrupted sutures technique |
| **C**omparison | Continuous closure technique |
| **O**utcome | Burst abdomen, incisional hernia, SSO and pain |

PICO 3.9 Closure technique in midline incision, small bites vs large bites technique

| **P**atient | Adults that underwent emergency surgery through a midline incision |
| --- | --- |
| **I**ntervention | Small bites |
| **C**omparison | Large bites |
| **O**utcome | Burst abdomen, incisional hernia, SSO and pain |

PICO 3.10 Suture material in midline incision

| **P**atient | Adults that underwent emergency surgery through a midline incision |
| --- | --- |
| **I**ntervention | Fast-absorbable suture material |
| **C**omparison | Slowly-absorbable suture material |
| **O**utcome | Burst abdomen, incisional hernia, SSO and pain |

PICO 3.11 Suture material in midline incision

| **P**atient | Adults that underwent emergency surgery through a midline incision |
| --- | --- |
| **I**ntervention | Non-absorbable suture material |
| **C**omparison | Slowly-absorbable suture material |
| **O**utcome | Burst abdomen, incisional hernia, SSO and pain |

PICO 3.12 Suture material in midline incision

| **P**atient | Adults that underwent emergency surgery through a midline incision |
| --- | --- |
| **I**ntervention | Antimicrobial impregnated sutures |
| **C**omparison | Not impregnated sutures |
| **O**utcome | Burst abdomen, incisional hernia, SSO |

PICO 3.13 Suture material in midline incision

| **P**atient | Adults that underwent emergency surgery through a midline incision |
| --- | --- |
| **I**ntervention | Monofilament sutures |
| **C**omparison | Multifilament sutures |
| **O**utcome | Burst abdomen, incisional hernia, SSO |

PICO 3.14 Interrupted absorbable vs continuous non/slowly-absorbable

| **P**atient | Adults that underwent emergency surgery through a midline incision |
| --- | --- |
| **I**ntervention | Interrupted stitches with fast-absorbable sutures |
| **C**omparison | Continuous stitches with slowly/non-absorbable sutures |
| **O**utcome | Burst abdomen, incisional hernia, SSO |

KQ3 – obesity - What is the preferred suture type and closure technique for a midline laparotomy in an **obese adult**?

PICO 3.15 Closure technique in midline incision, interrupted vs continuous

| **P**atient | Obese adults that underwent surgery through a midline incision |
| --- | --- |
| **I**ntervention | Interrupted sutures technique |
| **C**omparison | Continuous closure technique |
| **O**utcome | Burst abdomen, incisional hernia, SSO and pain |

PICO 3.16 Closure technique in midline incision, small bites vs large bites technique

| **P**atient | Obese adults that underwent surgery through a midline incision |
| --- | --- |
| **I**ntervention | Small bites |
| **C**omparison | Large bites |
| **O**utcome | Burst abdomen, incisional hernia, SSO and pain |

PICO 3.17 Suture material in midline incision

| **P**atient | Obese adults that underwent surgery through a midline incision |
| --- | --- |
| **I**ntervention | Fast-absorbable suture material |
| **C**omparison | Slowly-absorbable suture material |
| **O**utcome | Burst abdomen, incisional hernia, SSO and pain |

PICO 3.18 Suture material in midline incision

| **P**atient | Obese adults that underwent surgery through a midline incision |
| --- | --- |
| **I**ntervention | Non-absorbable suture material |
| **C**omparison | Slowly-absorbable suture material |
| **O**utcome | Burst abdomen, incisional hernia, SSO and pain |

PICO 3.19 Suture material in midline incision

| **P**atient | Obese adults that underwent surgery through a midline incision |
| --- | --- |
| **I**ntervention | Antimicrobial impregnated sutures |
| **C**omparison | Not impregnated sutures |
| **O**utcome | Burst abdomen, incisional hernia, SSO |

PICO 3.20 Suture material in midline incision

| **P**atient | Obese adults that underwent surgery through a midline incision |
| --- | --- |
| **I**ntervention | Monofilament sutures |
| **C**omparison | Multifilament sutures |
| **O**utcome | Burst abdomen, incisional hernia, SSO |

PICO 3.21 Interrupted absorbable vs continuous non/slowly-absorbable

| **P**atient | Obese adults that underwent surgery through a midline incision |
| --- | --- |
| **I**ntervention | Interrupted stitches with fast-absorbable sutures |
| **C**omparison | Continuous stitches with slowly/non-absorbable sutures |
| **O**utcome | Burst abdomen, incisional hernia, SSO |

KQ3 – non-midline - What is the preferred suture type and closure technique for a non-midline laparotomy?

PICO 3.22 Closure technique in non-midline incision (transverse/subcostal, paramedian and pfannenstiel), interrupted vs continuous

| **P**atient | Adults that underwent surgery through a non-midline incision |
| --- | --- |
| **I**ntervention | Interrupted sutures technique |
| **C**omparison | Continuous closure technique |
| **O**utcome | Burst abdomen, incisional hernia, SSO and pain |

PICO 3.23 Suture material in non-midline incision

| **P**atient | Adults that underwent surgery through a non-midline incision |
| --- | --- |
| **I**ntervention | Fast-absorbable suture material |
| **C**omparison | Slowly-absorbable suture material |
| **O**utcome | Burst abdomen, incisional hernia, SSO and pain |

PICO 3.24 Suture material in non-midline incision

| **P**atient | Adults that underwent surgery through a non-midline incision |
| --- | --- |
| **I**ntervention | Non-absorbable suture material |
| **C**omparison | Slowly-absorbable suture material |
| **O**utcome | Burst abdomen, incisional hernia, SSO and pain |

PICO 3.25 Suture material in non-midline incision

| **P**atient | Adults that underwent surgery through a non-midline incision |
| --- | --- |
| **I**ntervention | Antimicrobial impregnated sutures |
| **C**omparison | Not impregnated sutures |
| **O**utcome | Burst abdomen, incisional hernia, SSO |

PICO 3.26 Suture material in non-midline incision

| **P**atient | Adults that underwent surgery through a non-midline incision |
| --- | --- |
| **I**ntervention | Monofilament sutures |
| **C**omparison | Multifilament sutures |
| **O**utcome | Burst abdomen, incisional hernia, SSO |

PICO 3.27 Interrupted absorbable vs continuous non/slowly-absorbable

| **P**atient | Adults that underwent surgery through a non-midline incision |
| --- | --- |
| **I**ntervention | Interrupted stitches with fast-absorbable sutures |
| **C**omparison | Continuous stitches with slowly/non-absorbable sutures |
| **O**utcome | Burst abdomen, incisional hernia, SSO |

KQ4 Which patients have an increased risk of incisional hernia development?

KQ5 Is mesh augmentation beneficial during closure of **elective** laparotomies?

PICO 5.1 Mesh augmentation after midline incision

| **P**atient | Adults that underwent surgery through a midline incision |
| --- | --- |
| **I**ntervention | Mesh augmentation |
| **C**omparison | Primary closure |
| **O**utcome | Burst abdomen, incisional hernia, SSO, pain, mesh infection |

PICO 5.2 Mesh augmentation after non-midline incision

| **P**atient | Adults that underwent surgery through a non-midline incision |
| --- | --- |
| **I**ntervention | Mesh augmentation |
| **C**omparison | Primary closure |
| **O**utcome | Burst abdomen, incisional hernia, SSO, pain, mesh infection |

PICO 5.3 Mesh augmentation with permanent of absorbable mesh

| **P**atient | Adults that underwent surgery through midline incision |
| --- | --- |
| **I**ntervention | Mesh augmentation with absorbable mesh |
| **C**omparison | Mesh augmentation with permanent mesh |
| **O**utcome | Burst abdomen, incisional hernia, SSO, pain, mesh infection |

PICO 5.4 Mesh augmentation in different planes

| **P**atient | Adults that underwent surgery through midline incision |
| --- | --- |
| **I**ntervention | Mesh augmentation with an onlay mesh |
| **C**omparison | Mesh augmentation with a retromusculair mesh |
| **O**utcome | Burst abdomen, incisional hernia, SSO, pain, mesh infection |

PICO 5.5 Mesh augmentation in different planes

| **P**atient | Adults that underwent surgery through midline incision |
| --- | --- |
| **I**ntervention | Mesh augmentation with an onlay mesh |
| **C**omparison | Mesh augmentation with an intraperitoneal mesh |
| **O**utcome | Burst abdomen, incisional hernia, SSO, pain, mesh infection |

PICO 5.6 Mesh augmentation in different planes

| **P**atient | Adults that underwent surgery through midline incision |
| --- | --- |
| **I**ntervention | Mesh augmentation with an retromuscular mesh |
| **C**omparison | Mesh augmentation with an intraperitoneal mesh |
| **O**utcome | Burst abdomen, incisional hernia, SSO, pain, mesh infection |

KQ5 – emergency - Is mesh augmentation beneficial during closure of **emergency** laparotomies?

PICO 5.7 Mesh augmentation after midline incision

| **P**atient | Adults that underwent emergency surgery through a midline incision |
| --- | --- |
| **I**ntervention | Mesh augmentation |
| **C**omparison | Primary closure |
| **O**utcome | Burst abdomen, incisional hernia, SSO, pain, mesh infection |

PICO 5.8 Mesh augmentation with permanent of absorbable mesh

| **P**atient | Adults that underwent emergency surgery through midline incision |
| --- | --- |
| **I**ntervention | Mesh augmentation with absorbable mesh |
| **C**omparison | Mesh augmentation with permanent mesh |
| **O**utcome | Burst abdomen, incisional hernia, SSO, pain, mesh infection |

PICO 5.9 Mesh augmentation in different planes

| **P**atient | Adults that underwent emergency surgery through midline incision |
| --- | --- |
| **I**ntervention | Mesh augmentation with an onlay mesh |
| **C**omparison | Mesh augmentation with a retromusculair mesh |
| **O**utcome | Burst abdomen, incisional hernia, SSO, pain, mesh infection |

PICO 5.10 Mesh augmentation in different planes

| **P**atient | Adults that underwent emergency surgery through midline incision |
| --- | --- |
| **I**ntervention | Mesh augmentation with an onlay mesh |
| **C**omparison | Mesh augmentation with an intraperitoneal mesh |
| **O**utcome | Burst abdomen, incisional hernia, SSO, pain, mesh infection |

PICO 5.11 Mesh augmentation in different planes

| **P**atient | Adults that underwent emergency surgery through midline incision |
| --- | --- |
| **I**ntervention | Mesh augmentation with an retromuscular mesh |
| **C**omparison | Mesh augmentation with an intraperitoneal mesh |
| **O**utcome | Burst abdomen, incisional hernia, SSO, pain, mesh infection |

KQ6 Are postoperative abdominal binders advantageous after open abdominal surgery?

| **P**atient | Adults that underwent surgery through an abdominal wall incision |
| --- | --- |
| **I**ntervention | Postoperative abdominal binder |
| **C**omparison | No binder |
| **O**utcome | Burst abdomen, incisional hernia, SSO, pain, pulmonary complications |

KQ 7 Is restriction of activity advantageous after open abdominal surgery?

| **P**atient | Adults that underwent surgery through an abdominal wall incision |  |
| --- | --- | --- |
| **I**ntervention | Postoperative restriction of activities |  |
| **C**omparison | No restrictions |  |
| **O**utcome | Burst abdomen, incisional hernia, SSO, pain, pulmonary complications |  |

**Appendix S2. Complete search strategy and search terms for key question 1**

**Embase.com**

(laparotomy/mj OR laparoscopy/mj/exp OR 'abdominal surgery'/mj OR 'abdominal wall closure'/mj OR 'biliary tract surgery'/mj/exp OR 'gastrointestinal surgery'/mj/exp OR 'laparoscopy'/mj/exp OR 'laparotomy'/mj/exp OR 'liver surgery'/mj/exp OR 'omentectomy'/mj/exp OR 'omentoplasty'/mj/exp OR 'peritoneum lavage'/mj/exp OR 'spleen surgery'/mj/exp OR celioscopy/mj OR 'aneurysm surgery'/mj OR 'urologic surgery'/mj/exp OR (laparotom* OR laparoscop* OR laparoendoscop* OR celioscop* OR ((abdom* OR intraabdom* OR billiar*-tract* OR gastrointest* OR liver OR spleen OR bariatric* OR colorectal* OR pancrea* OR aneurysm* OR urolog* OR gynecolog* OR gynaecolog*) NEAR/3 (surg* OR incision OR closure OR resection* OR operat*)) OR pancreaticoduodenectom* OR pancreatoduodenectom* OR appendicectom* OR appendectom* OR gastrectom* OR proctectom* OR prostatectom* OR splenectom* OR hepatectom* OR colectom* OR (gastric NEAR/3 (bypass OR sleeve)) OR cholecystectom* OR nephrectom* OR cesarian* OR caesarian* OR hysterectom* OR oophorectom* OR salpingectom*):ti) AND ('incisional hernia'/de OR 'abdominal wall hernia'/de OR (hernia* OR (burst NEAR/3 (abdomen* OR abdomin*))):Ab,ti ) AND ('Controlled clinical trial'/exp OR 'Crossover procedure'/de OR 'Double-blind procedure'/de OR 'Single-blind procedure'/de OR (random* OR factorial* OR crossover* OR (cross NEXT/1 over*) OR placebo* OR ((doubl* OR singl*) NEXT/1 blind*) OR assign* OR allocat* OR volunteer* OR trial OR groups):ab,ti,kw) NOT ([animals]/lim NOT [humans]/lim) AND [2013-2021]/py NOT (juvenile/exp NOT adults/exp) NOT ([animals]/lim NOT [humans]/lim) NOT [conference abstract]/lim NOT (Gastroschisis/mj OR omphalocele/mj OR herniorrhaphy/mj OR (Gastroschisis* OR omphalocele* OR herniorrhaph* OR (abdom*-wall NEAR/3 reconstruct*)):ti) NOT [chinese]/lim AND ('midline incision'/de OR (midline OR periumbilical* OR peri-umbilical* OR (median NEAR/3 (incision OR laparotom*))):Ab,ti) AND ('transverse incision'/de OR (transverse OR Pfannenstiel OR Paramedian OR subcostal OR nonmedian OR non-median OR nonmidline OR non-midline OR oblique):ab,ti)

**Medline ALL Ovid**

(*Laparotomy/ OR exp *Laparoscopy/ OR exp *Biliary Tract Surgical Procedures/ OR exp *Digestive System Surgical Procedures/ OR *liver/su OR *spleen/su OR exp *Urologic Surgical Procedures/ OR (laparotom* OR laparoscop* OR laparoendoscop* OR celioscop* OR ((abdom* OR intraabdom* OR billiar*-tract* OR gastrointest* OR liver OR spleen OR bariatric* OR colorectal* OR pancrea* OR aneurysm* OR urolog* OR gynecolog* OR gynaecolog*) ADJ3 (surg* OR incision OR closure OR resection* OR operat*)) OR pancreaticoduodenectom* OR pancreatoduodenectom* OR appendicectom* OR appendectom* OR gastrectom* OR proctectom* OR prostatectom* OR splenectom* OR hepatectom* OR colectom* OR (gastric ADJ3 (bypass OR sleeve)) OR cholecystectom* OR nephrectom* OR cesarian* OR caesarian* OR hysterectom* OR oophorectom* OR salpingectom*).ti.) AND (Incisional Hernia/ OR Hernia, Abdominal/ OR (hernia* OR (burst ADJ3 (abdomen* OR abdomin*))).ab,ti. ) AND (Exp Controlled clinical trial/ OR "Double-Blind Method"/ OR "Single-Blind Method"/ OR "Random Allocation"/ OR (random* OR factorial* OR crossover* OR cross over* OR placebo* OR ((doubl* OR singl*) ADJ blind*) OR assign* OR allocat* OR volunteer* OR trial OR groups).ab,ti,kf.) AND (2013 OR 2014 OR 2015 OR 2016 OR 2017 OR 2018 OR 2019 OR 2020 OR 2021).yr. NOT (juvenile/ NOT adults/) NOT (exp animals/ NOT humans/) NOT (*Gastroschisis/ OR * Hernia, Umbilical/ OR * Herniorrhaphy/ OR (Gastroschisis* OR omphalocele* OR herniorrhaph* OR (abdom*-wall ADJ3 reconstruct*)).ti.) NOT Chinese.la. AND ((midline OR periumbilical* OR peri-umbilical* OR (median ADJ3 (incision OR laparotom*))).ab,ti.) AND ((transverse OR Pfannenstiel OR Paramedian OR subcostal OR nonmedian OR non-median OR nonmidline OR non-midline OR oblique).ab,ti.)

**Cochrane CENTRAL**

((laparotom* OR laparoscop* OR laparoendoscop* OR celioscop* OR ((abdom* OR intraabdom* OR billiar* NEXT tract* OR gastrointest* OR liver OR spleen OR bariatric* OR colorectal* OR pancrea* OR aneurysm* OR urolog* OR gynecolog* OR gynaecolog*) NEAR/3 (surg* OR incision OR closure OR resection* OR operat*)) OR pancreaticoduodenectom* OR pancreatoduodenectom* OR appendicectom* OR appendectom* OR gastrectom* OR proctectom* OR prostatectom* OR splenectom* OR hepatectom* OR colectom* OR (gastric NEAR/3 (bypass OR sleeve)) OR cholecystectom* OR nephrectom* OR cesarian* OR caesarian* OR hysterectom* OR oophorectom* OR salpingectom*):ti) AND ((hernia* OR (burst NEAR/3 (abdomen* OR abdomin*))):Ab,ti ) AND ((random* OR factorial* OR crossover* OR (cross NEXT/1 over*) OR placebo* OR ((doubl* OR singl*) NEXT/1 blind*) OR assign* OR allocat* OR volunteer* OR trial OR groups):ab,ti,kw) NOT ((Gastroschisis* OR omphalocele* OR herniorrhaph* OR (abdom* NEXT wall NEAR/3 reconstruct*)):ti) AND ((midline OR periumbilical* OR peri NEXT umbilical* OR (median NEAR/3 (incision OR laparotom*))):Ab,ti) AND ((transverse OR Pfannenstiel OR Paramedian OR subcostal OR nonmedian OR non NEXT median OR nonmidline OR non NEXT midline OR oblique):ab,ti)
